# Limitations of Deep Learning Attention Mechanisms in Clinical Research: Empirical Case Study Based on the Korean Diabetic Disease Setting

**DOI:** 10.2196/18418

**Published:** 2020-12-16

**Authors:** Junetae Kim, Sangwon Lee, Eugene Hwang, Kwang Sun Ryu, Hanseok Jeong, Jae Wook Lee, Yul Hwangbo, Kui Son Choi, Hyo Soung Cha

**Affiliations:** 1 Graduate School of Cancer Science and Policy National Cancer Center Goyang-si, Gyeonggi-do Republic of Korea; 2 Cancer Data Center National Cancer Control Institute National Cancer Center Goyang-si, Gyeonggi-do Republic of Korea; 3 Healthcare AI Team Healthcare Platform Center National Cancer Center Goyang-si, Gyeonggi-do Republic of Korea; 4 School of Management Engineering Korea Advanced Institute of Science and Technology Seoul Republic of Korea; 5 Division of Nephrology Department of Internal Medicine National Cancer Center Goyang-si, Gyeonggi-do Republic of Korea; 6 Division of Endocrinology Department of Internal Medicine National Cancer Center Goyang-si, Gyeonggi-do Republic of Korea

**Keywords:** attention, deep learning, explainable artificial intelligence, uncertainty awareness, Bayesian deep learning, artificial intelligence, health data

## Abstract

**Background:**

Despite excellent prediction performance, noninterpretability has undermined the value of applying deep-learning algorithms in clinical practice. To overcome this limitation, attention mechanism has been introduced to clinical research as an explanatory modeling method. However, potential limitations of using this attractive method have not been clarified to clinical researchers. Furthermore, there has been a lack of introductory information explaining attention mechanisms to clinical researchers.

**Objective:**

The aim of this study was to introduce the basic concepts and design approaches of attention mechanisms. In addition, we aimed to empirically assess the potential limitations of current attention mechanisms in terms of prediction and interpretability performance.

**Methods:**

First, the basic concepts and several key considerations regarding attention mechanisms were identified. Second, four approaches to attention mechanisms were suggested according to a two-dimensional framework based on the degrees of freedom and uncertainty awareness. Third, the prediction performance, probability reliability, concentration of variable importance, consistency of attention results, and generalizability of attention results to conventional statistics were assessed in the diabetic classification modeling setting. Fourth, the potential limitations of attention mechanisms were considered.

**Results:**

Prediction performance was very high for all models. Probability reliability was high in models with uncertainty awareness. Variable importance was concentrated in several variables when uncertainty awareness was not considered. The consistency of attention results was high when uncertainty awareness was considered. The generalizability of attention results to conventional statistics was poor regardless of the modeling approach.

**Conclusions:**

The attention mechanism is an attractive technique with potential to be very promising in the future. However, it may not yet be desirable to rely on this method to assess variable importance in clinical settings. Therefore, along with theoretical studies enhancing attention mechanisms, more empirical studies investigating potential limitations should be encouraged.

## Introduction

In recent years, there has been significant evidence that deep-learning algorithms can outperform other machine-learning algorithms and conventional statistics in the medical field [1,2]. Despite the better prediction accuracy than conventional algorithms, the implications of using deep learning have been limited owing to the inability to explain the models [3,4]. Particularly in the medical environment, where the association between a disease and symptoms must be identified to provide adequate treatments, the interpretability of models is very important [3-5]. To overcome these limitations, interpretable deep-learning algorithms such as Shapley Additive Explanations (SHAP), Local Interpretable Model-agnostic Explanations (LIME), and attention mechanisms have been introduced [6-8]. The commonality of these three methodologies provides interpretability in the form of variable importance [6-8]. The difference between the methodologies is that with SHAP or LIME, variable importance is measured through simulations that change the data after model training is completed [6,7], whereas under attention mechanisms, variable importance is inferred during model training, which improves model performance by weighting several important variables [8,9].

Based on this advantage, attention mechanisms have starting to gain appeal in the clinical research field [10-17]. However, there is a gap between the application of attention mechanisms in clinical research and up-to-date attention algorithms in development. Specifically, most of the recent attention studies have focused on improving the theoretical robustness, design approach, and model accuracy with attention mechanisms [10-17]. However, clinical researchers are more interested in potential limitations that may arise when attention mechanisms are applied, and how they may differ from conventional statistics, than in the details as to how robust and sophisticated attention mechanisms are being developed. A few studies have introduced the potential limitations of attention mechanisms [18,19]. However, these studies have been theoretical, making it difficult for clinical researchers to understand and accept the results. Thus, it is increasingly necessary to provide a discussion of what clinical researchers should be aware of when applying the new concept of attention mechanisms in their research.

With the goal of reducing this gap, the aim of this study was to evaluate attention mechanisms in terms of prediction performance and interpretability. In addition, there remains a lack of guidance for clinical researchers in the implementation of attention mechanisms; therefore, to facilitate understanding for clinical researchers, this study preemptively provides basic concepts, key considerations, and codes for attention mechanisms. Finally, a case analysis was performed in a cross-sectional and structured data environment, which is the simplest data setting possible for clinical researchers.

This study was conducted according to the following procedure. First, the scope of the study was established in terms of the data structure. Then, a brief introduction and several important considerations regarding attention mechanisms were considered. Second, based on previous research, a two-dimensional framework was established to guide the four modeling approaches to attention mechanisms. Third, five empirical tests with attention mechanisms were performed using the four models: prediction performance, probability reliability, concentration of variable importance, consistency of attention results, and generalizability of attention results to conventional statistics. Finally, potential limitations that may arise when using attention mechanisms were identified.

## Methods

### Research Scope

Since the design approaches of attention mechanisms differ greatly depending on the data structure, the scope of this study was established in terms of data structure. Specifically, attention mechanism research in the medical field can be divided into two main categories from a data point of view. The first category is an unstructured data area where data containing natural language and images cannot be stored in a row and column table structure [20,21]. In the field of natural language processing, attention mechanisms have been applied to determine the relationship between words or between words and diseases in clinical notes [13,14,22]. In the image area, attention mechanisms have been used to highlight which parts of clinical images were related to clinical events, or to annotate the images [15-17]. The second category is a structured data area where data can be organized in table formats with a row and column structure [21]. In this area, attention mechanisms have been applied to electronic health records to determine variables that are strongly associated with clinical events [9,12,23].

Structured data familiar to clinical researchers are widely applicable to most statistical analyses, including linear regression analysis and analysis of variance (ANOVA). Since one purpose of this study was to compare the results of attention mechanisms and conventional statistical methods, the scope of the study was limited to structured data. Furthermore, most previous attention mechanism studies using structured data have been conducted in time-series settings [9,12,23]. However, this study was conducted in a cross-sectional data setting, which is simpler and easier than a time-series data setting, and can therefore help readers less familiar with attention mechanisms to better understand the results of the case study.

### Introduction to Attention

#### Concepts of Attention Mechanisms

**Figure 1:**
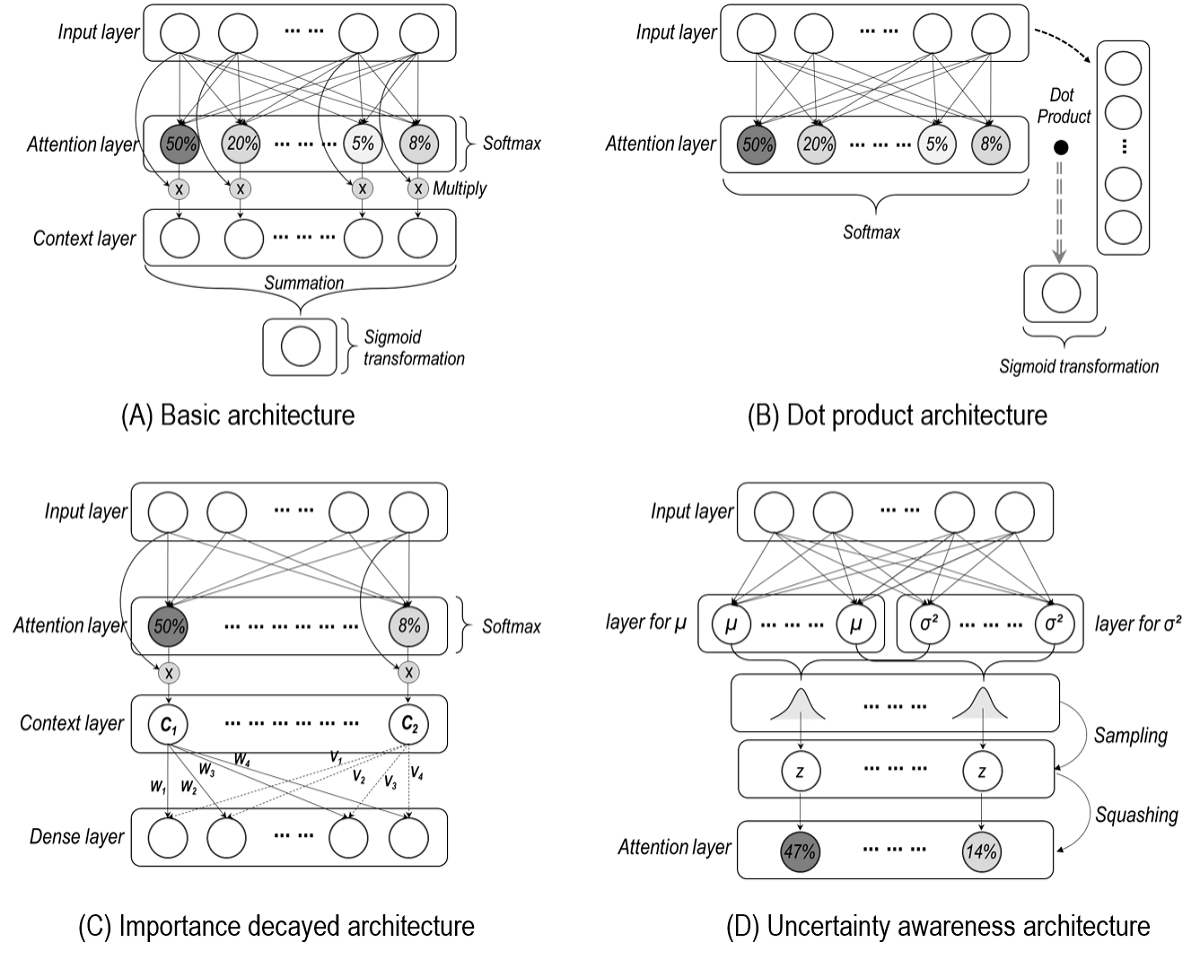
Model structures for attention implementation. (A) Basic architecture of an attention mechanism model. (B) Model architecture where the dot product is employed for transferring the influence of input variables toward the outcome. (C) Model architecture where the importance of input variables may be decayed. (D) Model architecture that is aware of uncertainty. The percentages in the circles show examples of attention values.

Attention, one of the layers in a neural network model, quantifies the importance of input variables in terms of their impact on outcomes (Figure 1) [8,24,25]. Attention is mostly calculated based on the Softmax function (Notation A1 in Multimedia Appendix 1), such that each node in the layer has a value between 0 and 1 and the sum of all node values must be 1 [8,24,25]. When the node size of attention is equal to the number of input variables, the influence of the input variables can be transferred toward the model outcome by multiplying the attention values with the corresponding input variables (Context layer in Figure 1A) [8,24,25]. Accordingly, in the case of binary classification, all values in the context layer are summed together to produce a single value (Summation in Figure 1A). The efficient model design without context layers is possible through the dot product between the input and attention values (Figure 1B). Finally, the single value may be converted to a value between 0 and 1 through the sigmoid function (Sigmoid transformation in Figure 1A and B).

The attention value of a certain variable indicates the relative importance of that variable compared with that of other variables. If the attention value of a particular variable is large, the large influence of that variable is transmitted when predicting the outcome variable. As an extreme example, when the attention value of a variable is 1, only that variable is used to predict the outcome variable, whereas if the attention value of a variable is 0, that variable is not used to predict the outcome variable. Figure 1A shows the basic architecture of a model with attention mechanisms; the code for the model implemented in Keras is provided in Codes A1 of Multimedia Appendix 2.

#### Consideration in Attention Modeling

Attention mechanisms can be implemented in various ways, because the key feature of deep-learning modeling is that users can freely design the structure [8,9,23-25]. However, there is a primary important consideration in implementing attention mechanisms. In some cases, the influence of variable importance in the context layer can be distorted. For instance, if all *w_i_* values are close to 0, the value of *C_1_* has a minor effect on the next layer even if that value is the highest in the context layer (Figure 1C). Moreover, even if the value of *C_2_* is the lowest, if all *v_i_*s have very large positive values, the large influence of *C_2_* can be passed to the next layer (Figure 1C). As such, context values can be skewed as they are computed through a weight matrix in the process of being passed to the next layer (ie, Dense layer in Figure 1C). As a result, the skewed effects can be propagated to the model output if the output is inferred from the layer. Therefore, it is very important to design a structure where the outputs are not computed through weight matrices [9,23].

### Modeling Approaches

Although deep-learning models can be developed in various ways depending on the tendency of developers, two approaches have been commonly applied in recent attention studies: increasing the degrees of freedom and uncertainty awareness (UA).

#### Increase in the Degrees of Freedom

The mechanism for increasing the degrees of freedom is to design multi-attention layers; representative algorithms that reflect such a mechanism include transformer and bidirectional transformer (BERT) [8,26]. Our intuition regarding the effectiveness of the mechanism relies on the idea that models can learn the importance of input variables from various perspectives [8,26]. Given the randomness feature of deep-learning training, the result from one attention layer can be unreliable. However, the multi-attention model offers multiple result sets with variable importance so that a reliable set of results may compensate for an unreliable set. Consequently, models in which multi-attention layers are applied have recently shown better performance than other models [8,26-28].

#### UA

Deep-learning algorithms are not free from the uncertainty issue, which concerns the fact that prediction results have the potential to be incomplete in terms of accuracy and consistency [9,29-33]. The major sources of uncertainty include data with noise and omissions, the complexity of the model associated with the parameters (ie, number of weights and type of activation functions), and the structures (ie, degree of depth) [30,31]. One way to alleviate this issue is to consider the presence of uncertainty in modeling [9,29,32,33]. Specifically, we may assume that node values (ie, attention values) in a certain layer come from a distribution with a mean (μ) and a variance (σ^2^; Figure 1D) [9,29,32,33]. A normal distribution (ie, a Gaussian distribution) that is theoretically clear and can be computed efficiently is often assumed [9,29,32,33]. Based on this assumption, certain values with high probability are estimated, which may mitigate the random nature of deep-learning training [9,29-33]. A representative model designed under these assumptions is the variational auto-encoder [32,33].

### Framework for Empirical Tests

**Figure 2:**
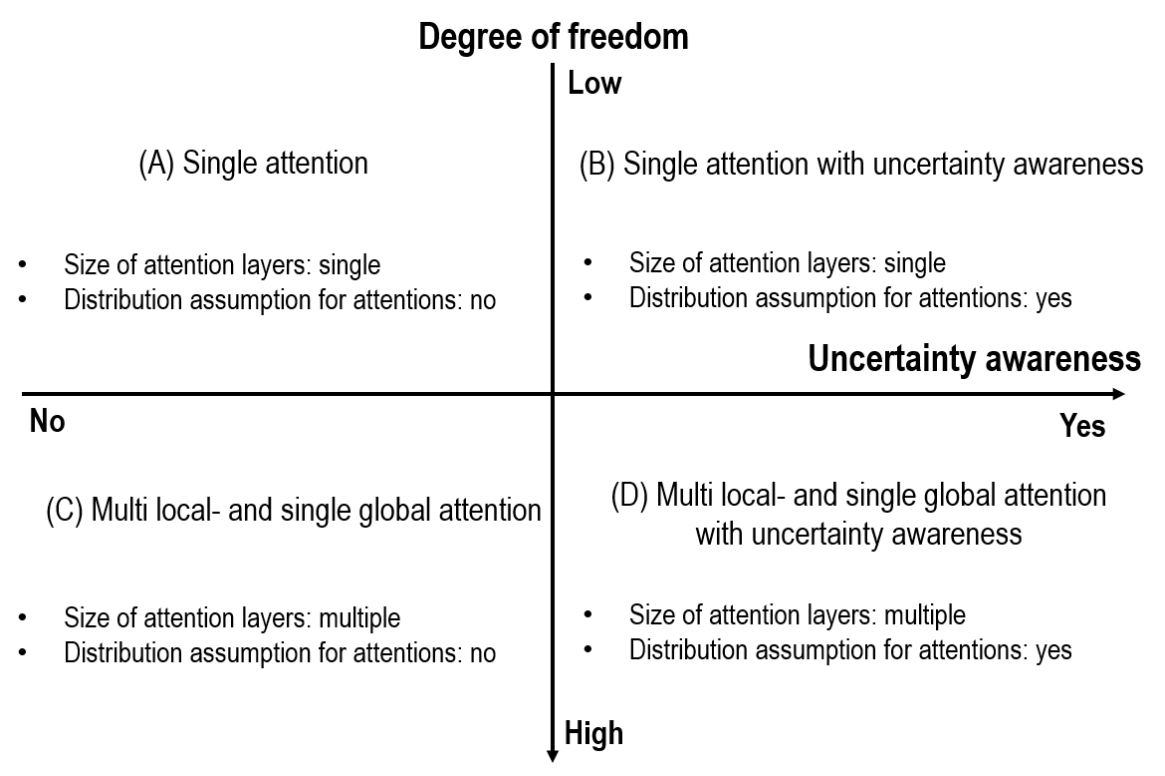
Framework for empirical tests.

Based on the discussion above, two directions (ie, degree of freedom and UA) were considered for attention modeling (Figure 2). In this two-dimensional framework, four cases were suggested for empirical tests (Figure 2). Degree of freedom is related to model structures and UA is related to the estimation approach.

Empirical test entries for the four models in the framework were categorized into two broad categories: outcome and attention (Table 1). In terms of model outcome, a receiver operating characteristic (ROC) test, which expresses model accuracy based on the relationship between sensitivity and specificity, was employed for prediction performance [34]. In addition, the performance of probability reliability, which measures the degree of agreement between predicted and actual probability, was assessed using a reliability diagram and Brier scores [35-37].

In terms of attention, the degree of how concentrated the variable importance was in particular variables was measured (ie, Concentration in Table 1). The Herfindahl index, which represents the degree of concentration with values ranging from near 0 (least concentrated) to 1 (most concentrated), was employed for this measure [38]. Furthermore, correlation analysis was conducted to evaluate the consistency of attention results between multiple instances. Lastly, the generalizability of attention results was tested in two ways. First, the variable effect sizes obtained from conventional statistical methods (*t* test, Cohen *d*; chi-square test, Cramer *V*) were compared with variable importance (ie, attention values) [39,40]. For clear comparison from a clinical point of view, only the top 5% of the variables in terms of effect size (ie, conventional methods) and variable importance (ie, attention) were compared. Second, regression analysis was used to determine the overall relationship between attention values and effect sizes from conventional methods.

**Table 1:** Empirical test entries for measuring the performance of four models.

Entries (measures)		Methods
**Outcome**		
	Prediction performance	Receiver operating characteristic
	Probability reliability	Reliability diagrams
**Attention**		
	Concentration	Herfindahl index (near 0, least concentrated; 1, most concentrated)
	Consistency	Correlation
	Generalizability	Effect size: Cohen *d* (*t* test), Cramer *V* (chi-square test) Regression analysis (dependent variable, effect size obtained from conventional methods; independent variable, attention values)

### Model Specifications

#### Model Designs

**Figure 3:**
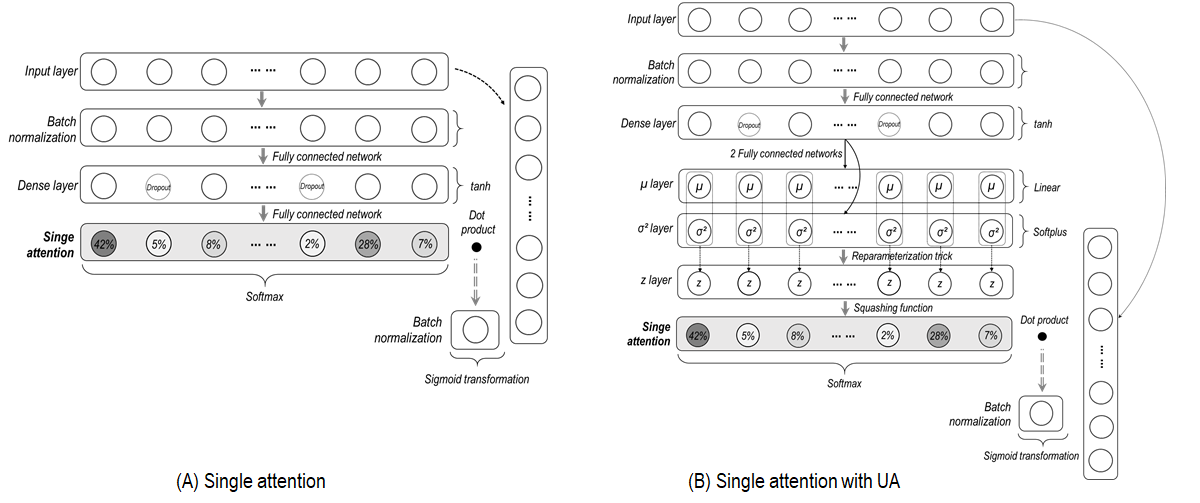
Model designs for single attention mechanisms. UA: uncertainty awareness. The concept of "reparameterization trick" is described in Concept A1 of Multimedia Appendix 1.

**Figure 4:**
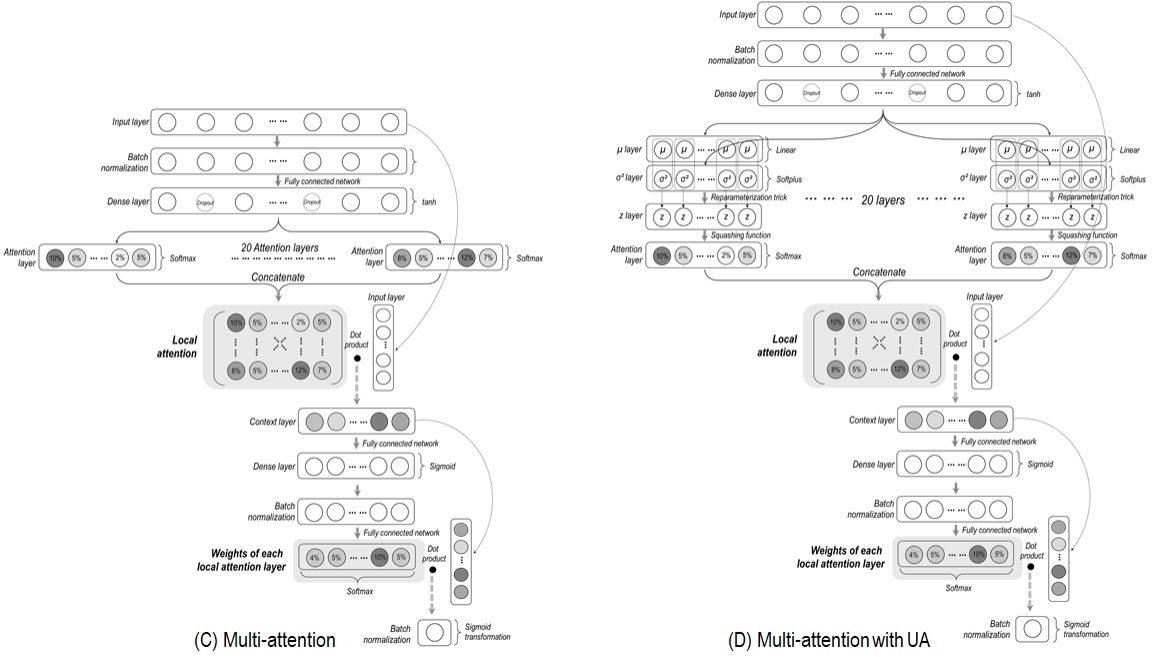
Model designs for multi-attention mechanisms. UA: uncertainty awareness. The concept of "reparameterization trick" is described in Concept A1 of Multimedia Appendix 1.

Four models were developed according to the framework presented in Figure 2. The letters A, B, C, and D represent quadrants on the framework that correspond to the letters representing the model designs in Figure 3 and Figure 4. Model A (without any uncertainty considerations) has only a single attention layer (Figure 3A). The basic design of model B is the same as that of model A; however, it differs in that it has additional layers for UA (see layers with *μ*, *σ^2^*, and *z* in Figure 3B). Thus, attention values in model B were estimated from the Gaussian distribution [9,33].

The two models in which the degree of freedom is considered are presented in Figure 4. The difference between models C and D is that uncertainty in attention estimation is considered in model D (see layers with *μ, σ^2^*, and *z* in Figure 4D).

Since these models have multi-attention layers (ie, Local attention in Figure 4) with a heuristic size of 20, multiple attentions are estimated. Thus, a novel structure was designed to convey the multiple values in the direction of model outcomes. Specifically, a context layer was created as the dot product of the local attention layer and the input layer (see Context layer in Figure 4). Each value on the context layer represents the summed impact of each corresponding local attention layer. Next, a “Weights of each local attention layer” was formed, whose role is to weigh (with weights between 0 and 1) the summed impact values in the context layer (Figure 4). Lastly, the outcome layer was created as the dot product of the weights of each local attention layer and the context layer.

This somewhat complex structure ensures that the influence of one variable is passed only once to the model outcome, even if attention values are inferred multiple times (20 times in this case). Furthermore, using both the local attention layer and the weights corresponding to each vector, a unique attention value for each variable can be obtained, which facilitates interpretation.

Graphical and mathematical notations are provided for obtaining a set of unique values (global attention in Figure A1 of Multimedia Appendix 1). In addition, details of the four models are provided as Keras codes in Codes A2-A5 of Multimedia Appendix 2.

#### Settings for Rigorous Analysis

A 10-fold test was performed to assess the empirical test entries. The dataset was divided into 10 test sets (10% of total sets) and 10 training sets (90% of total sets). The training sets were then subdivided with 80% used directly for model training and 20% for validation.

Entries related to the model outcome (Table 1) were evaluated using all predicted probabilities of the entire sample. In other words, all of the values estimated from the 10 test sets were combined into one total set, which was then used for testing. Entries related to model attention were assessed based on the level of fold sets. Specifically, all of the estimated attention values were aggregated so that each set of 10 folds had a representative value. For rigorous estimation, both outcome and attention were estimated through Monte Carlo simulations with 100 trials [5,25]. Detailed procedures for estimating outcome and attention values are provided in the form of pseudoalgorithms (Algorithms A1-A3) in Multimedia Appendix 2.

#### Cost Functions

The binary cross-entropy function, which is generally used in binary outcome settings [41,42], was employed for models A and C where UA was not considered. However, the loss functions for models B and D, given their UA, were specified differently.

The UA models assume that the model outcome is dependent on the normal distribution (ie, layers with *μ, σ^2^*, and *z*), which infers the attention value [9,32,33]. Therefore, the distribution associated with attention should be considered in the cost function. The cost function under these assumptions was derived through Bayesian inference theory [9,32,33,43]. According to this theory, the network weights should be learned so that the distributions in the *z* layer generated by the weights (see *z* layer in Figure 3B) become similar to the true distributions in the *z* layer [9,32,33,43]. Therefore, the cost function for uncertainty awareness models consists of two terms: the loss associated with the model outcome and the degree of similarity associated with the *z* distribution [9,32,33,43]. The cost function, with its description, is presented in Notation A2 of Multimedia Appendix 1.

#### Learning Environments and Parameters

Attention models were developed, learned, and tested on Keras 2.3.1, tensorflow 2.1.0, and Python 3.7.6. Adam with a learning rate of 0.001 was employed as an optimizer to train all models. A training dataset with a batch size of 5000 was provided to the model. The early-stop rule was applied to stop training the models at the optimal epoch. Thus, model training was terminated when the loss value of the validation set did not improve further during the last 1200 epochs. Other details about activation functions and the structure of nodes and layers are provided in Code A2 to Code A5 of Multimedia Appendix 2. For the effect sizes of conventional statistical methods, the values for Cohen *d* and Cramer *V* were obtained from researchpy 0.2.3, a third-party Python library. Additionally, regression analysis was performed on Stata 13, a commercial statistical analysis software.

### Data

The case analysis was performed in a setting where the relationship between a disease and other variables is well established: an 8-year (2010 to 2017 inclusive) cumulative Korea National Health and Nutrition Examination Survey dataset, which assesses the nutrition and health status of Koreans and collects information about major chronic diseases such as metabolic syndrome and diabetes [44]. Since the association between diabetes and other variables has been well established through prior studies using these data, this selection facilitated a clear assessment of the empirical test results of this study [45-49]. Thus, a diabetes diagnosis (1=diabetes, 0=no diabetes) was set as the outcome variable for the four attention models. The subjects were classified as having or not having diabetes based on whether they were diagnosed by a doctor, or received diabetes medication or insulin injections. Fasting blood glucose levels, which are a very strong indicator for diagnosing diabetes, were intentionally used as an input variable to evaluate the power of the attention mechanism for determining important variables.

In the 8-year cumulative data, only variables with consistent labels during that period were included. Variables with no change in value, containing more than a 50% omission rate and subject identification information were excluded from the study set. Categorical variables of both nominal and ordinal types were integerized using integer encoding [50]. In other words, class labels of each categorical variable were converted into integers. Missing values were encoded as the extreme value 99,999. Since deep-learning algorithms can learn the nonlinear relations among variables [51], these encoding approaches can be workable and are efficient in settings where preprocessing is demanding owing to many variables. All values in input variables except for a missing value indicator (ie, 99,999) were normalized to be between 0 and 1, and were then fed into the deep-learning models.

## Results

### Data Preprocessing

There were 238 variables with consistent labels in the 8-year cumulative dataset. Only 128 variables were selected by preprocessing. There were 22 variables with no change in value, 84 variables with more than 50% missing values, and 4 variables containing identification information that were excluded from the analysis. The total number of observations (ie, the number of subjects) was 33,065, with an average age of 48.89 years and with men accounting for 40.41% (n=13,361) of the sample. Only 6 variables had no omissions, and the average missing rate of variables with omissions was 10.38%

### Outcome

#### Prediction Performance

**Figure 5:**
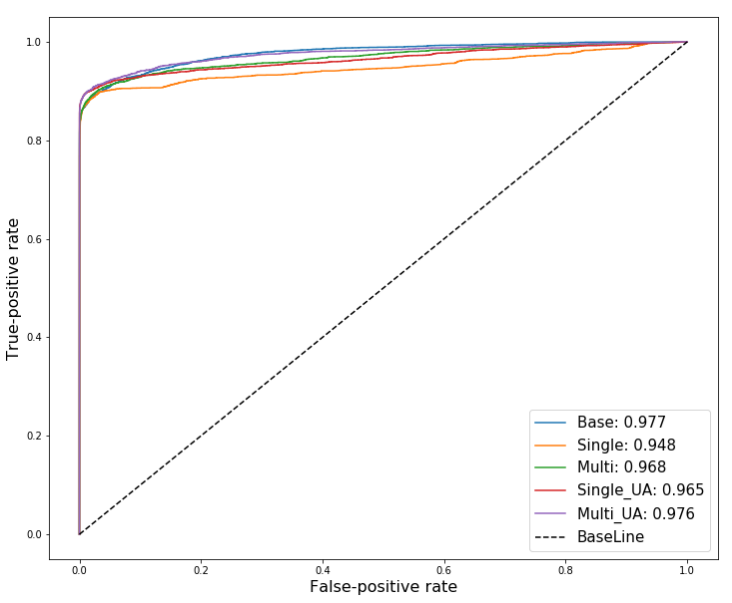
Receiver operating characteristic (ROC) test for the four models. All values estimated from 10 test sets are combined into one set for each model. The base indicates the deep-learning model without attention mechanisms. UA: uncertainty awareness.

Figure 5 represents the results for the ROC test and area under the curve (AUC) values of the five models. The results are based on the combined sets of predicted probabilities of 10 test sets of each model. According to the AUC results, the accuracy of the five models in terms of sensitivity and specificity was excellent overall. The AUCs of the base model without the attention mechanism, the single attention model, multi-attention model, single attention model with UA, and multi-attention model with UA were 0.977, 0.948, 0.968, 0.965, and 0.976, respectively.

#### Probability Reliability

**Figure 6:**
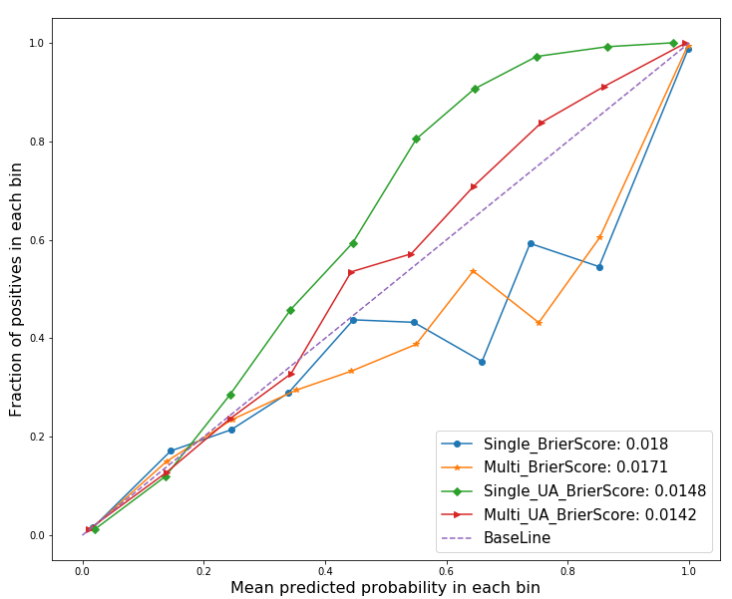
Reliability diagrams for four models. The Brier score measures the overall reliability of probabilistic predictions. UA: uncertainty awareness.

Figure 6 shows the performance of probability reliability for the four models in the form of a reliability diagram. A characteristic of the UA models is that most fractions of positives were plotted above the diagonal. By contrast, models without UA showed more fractions of positives below the diagonal than the other models. The fraction of positives of the multi-attention with UA model was the closest to the diagonal. The Brier scores of the single attention, multi-attention, single attention with UA, and multi-attention with UA models were 0.018, 0.0171, 0.0148, and 0.0142, respectively.

### Attention

#### Concentration

**Figure 7:**
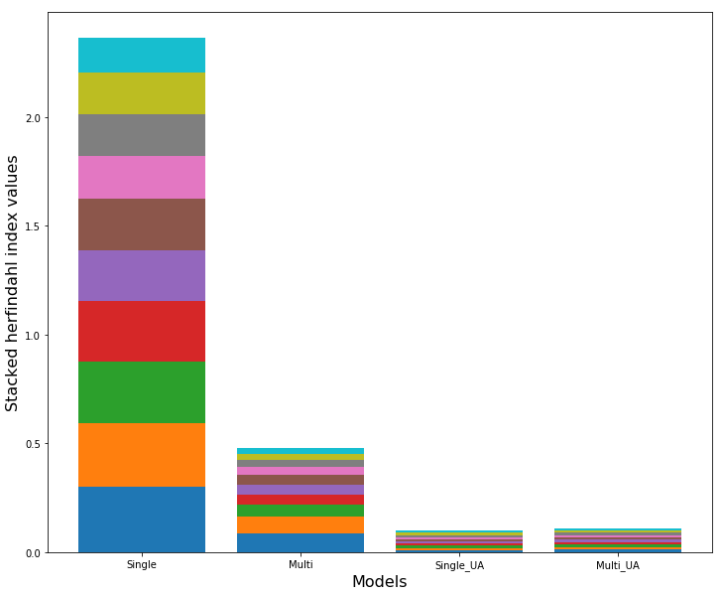
Herfindahl index values of 10-fold sets for each model. UA: uncertainty awareness.

Figure 7 shows stacked Herfindahl indices sorted by value size for the 10-fold sets in each model. The values for each fold are presented in Table A1 of Multimedia Appendix 1. In general, models without UA showed relatively large Herfindahl indices. The average Herfindahl index values for the single attention and multi-attention models were 0.236 and 0.048, respectively. However, models with UA had very small values, regardless of the degree of freedom. The average Herfindahl index values for the single attention with UA and multi-attention with UA models were 0.01 and 0.01, respectively. These results indicate that influence is more concentrated on several variables in models where uncertainty is not considered than in those where uncertainty is considered.

#### Consistency

**Figure 8:**
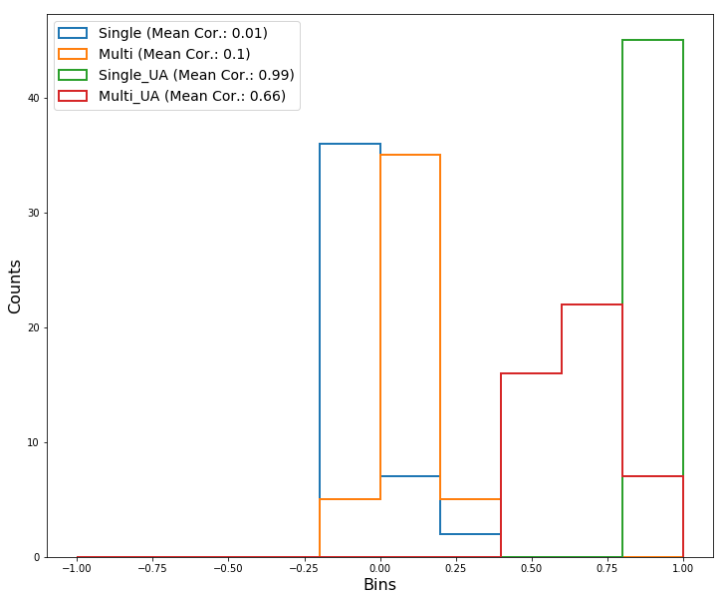
Histograms for correlations among 10-fold sets for each model. UA: uncertainty awareness.

Figure 8 shows histograms for the 45 correlations ({[10×10]–10}/2) among the 10-fold sets for each model. In general, the correlations of the fold sets from the models with UA were higher than those of the models without UA. The average correlations of the fold sets from both models without UA were calculated to be close to zero (ie, 0.01 and 0.1). Moreover, the average correlations from the two models with UA were calculated as 0.99 and 0.66, respectively.

#### Generalizability

Table 2 shows the results of the variable importance learned by the attention models and the effect sizes measured by conventional statistical methods. Definitions of each variable are provided in Table A2 of Multimedia Appendix 1. The top 5% (127×0.05=6) of variables, sorted by the magnitude of values obtained from each method, are reported. Since Cohen *d* from conventional methods may take on negative values, the absolute value was applied when sorting. Overall, models in which uncertainty was not considered were trained to have high attention values. Furthermore, variables such as “allownc” (whether to receive a basic living allowance) and “house” (whether to have own house) that bear little relation to health status were included in the results.

**Table 2:** Top 5% variable importance results estimated by different methods.

Variable^a^		Attention value^b^/Effect size^c^	
**Single attention model**			
	sm_presnt		0.0615
	BD1		0.0519
	pa_walk		0.0479
	HE_Upro		0.0468
	HE_alt		0.0430
	Npins		0.0413
**Multi-attention model**			
	HE_HB		0.041
	Sex		0.033
	Pa_walk		0.032
	HE_HBsAg		0.027
	Allownc		0.026
	HE_sbp		0.026
**Single attention model with UA^d^**			
	pa_walk		0.050
	BH9_11		0.017
	HE_THfh2		0.011
	HE_THfh1		0.010
	HE_THfh3		0.010
	DI5_dg		0.010
**Multi-attention model with UA**			
	pa_walk		0.050
	HE_THfh2		0.019
	BH9_11		0.015
	HE_THfh1		0.013
	HE_ast		0.010
	house		0.010
**Conventional statistics^e^**			
	age		1.536
	HE_glu		1.214
	HE_HbA1c		–1.014
	Wt_pool_1		–0.516
	Wt_itvex		–0.516
	HE_Uglu		0.319

^a^See Table A2 in Multimedia Appendix 1 for variable label descriptions.

^b^Average from the 10 fold sets.

^c^Effect size is presented only for the conventional statistics.

^d^UA: uncertainty awareness.

^e^Null hypothesis of categorical variables=no relationships between diabetes and a categorical variable; null hypothesis of continuous variables=no difference in variables between the diabetes and no diabetes groups.

Table 3 shows the overall relationship between the effect sizes of variables and variable importance. Since attention values from both single attention and multi-attention models with UA had a high correlation (0.943), two regression models in which the two variables did not overlap were specified. The regression results showed no association between the variable importance from attention models and the effect size of variables from conventional methods.

**Table 3:** Regression analysis results for assessing an association between attention values and effect sizes.

Regression model variables^a^	Regression 1		Regression 2	
	Coefficient	*P* > *t*	Coefficient	*P* > *t*
Single attention	−0.696	0.635	−0.668	0.652
Multi-attention	1.897	0.487	2.019	0.476
Single attention with UA^b^	−1.351	0.797	—^c^	—
Multi-attention with UA	—	—	−1.541	0.772
Intercept	0.075	0.09	0.075	0.075

^a^The dependent variable is the absolute value of effect size, calculated by Cohen *d* for continuous variables and Cramer *V* for categorical variables. The total number of observations is equal to the number of variables.

^b^UA: uncertainty awareness.

^c^—: variable not included in the regression model.

## Discussion

### Principal Findings

#### Reliability

A difference in performance according to the degree of freedom was prominent in the probability reliability diagram (Figure 6). The fraction of positives located above the diagonal indicates that probabilities are predicted to be larger than expected, while the fraction of positives located below the diagonal means that probabilities are estimated to be smaller than expected [35,36]. In this regard, overall probabilities from the two attention models without UA tended to be underestimated, whereas the attention models with UA tended to overestimate probabilities. Although no clear causal relationship has been identified, several lines of empirical evidence suggest that the over- and underestimation is associated with data noise, estimation methods, and parameter settings [30,52-54].

Since the difference appeared to be based on the UA axis, the over- or underestimation tendency of the models may be related to UA. Furthermore, the Brier scores of the two models with UA were smaller than those of the two models without UA, indicating that models with UA tend to estimate more reliable probabilities than models without UA. These findings are consistent with the results of recent research that estimated reliable outcomes with an emphasis on UA [9,55-57]. Theoretically, the most probabilistic values are inferred from a distribution that takes means and variances into account under UA [9,55-57]. Thus, the awareness of uncertainty may bring reliability to the prediction results of deep-learning models, which are vulnerable to randomness during the learning process.

#### Consistency

UA produced noticeable differences in results consistency and the concentration of variable importance. Specifically, in UA models with low Herfindahl indices, variable importance appeared to be distributed over many variables in contrast to models that did not consider uncertainty (Figure 7). In addition, high correlations between 10-fold sets were found in the attention results from the UA models, whereas no correlations were found in the results from UA models (Figure 8). Furthermore, the attention values in the models with UA were generally smaller than those of models without UA.

These results suggest that the consistency of results from the UA models is high because the variable importance with overall low values is distributed evenly over most variables. This result is closely associated with the assumption that attention values were estimated based on a normal distribution within the cost function (see equation for the Kullback–Leibler divergence *D_KL_* in Notation A2 of Multimedia Appendix 1). According to this equation, as both *μ* and *σ^2^* approach zero, model parameters for forming the normal distribution approximate the true theoretical distribution, indicating that the models are well learned [32,33]. Consequently, the overall attention values were small since the overall values of *μ* were small.

#### Spurious Correlations

As with conventional statistical methods, the attention models were unable to control spurious correlations during attention learning. Specifically, of the top 5% of variables obtained from conventional statistics, wt_pool_1 (interview weight combined years) and wt_itvex (interview weight for a single year) have little to do with health status (Table 2). These variables are weights for compensating errors due to differences in the number of households and populations between the sample design time and the survey time. In addition, the variables “allownc” (whether to receive basic living allowance) and “house” (whether to have own house) were obtained from the attention models (Table 2). These results may suggest spurious correlations in the dataset itself [58]. In other words, these variables, with little relation to diabetes, have a relatively close relationship with diabetes only by chance.

#### Generalizability to Conventional Statistics

In terms of clinically relevant variables, no significant association between the results of conventional statistics and attention models was found. Specifically, the variables age, HE_glu (fasting blood sugar), HE_HbA1c (hemoglobin AIC), and HE_Uglu (urine glucose) selected by conventional methods are well known to have a direct association with diabetes [59-61]. In addition, several variables, including pa_walk (amount of walking) and BH9_11 (vaccination status against influenza virus), obtained by the attention models are less directly related to diabetes. These variables may represent behavioral characteristics of patients with diabetes who are trying to manage their health.

Furthermore, there was no intersection of variables selected by both attention models and conventional statistical methods (Table 2). In particular, HE_glu, which was intentionally used as an input variable for testing purposes, was not determined as a major variable in the attention mechanism models in contrast to the conventional statistical methods. Additionally, no variable was statistically significant in the regression analysis that evaluated the positive association between attention values and effect sizes (Table 3). Comprehensively, these results suggest that the variable importance obtained from attention mechanisms may not be generalized to the effect size of conventional statistics.

### Lessons from the Findings

#### Hyperparameters 

The model structure and weight of terms in the cost function are hyperparameters to be adjusted. In terms of the model structure, the degree of freedom of attention layers was evaluated by comparing two extreme cases of 1 attention layer and 20 attention layers. Although the size of attention layers does not make a significant difference, the results can be significantly different if the number of attention layers is different under other conditions.

Furthermore, by taking uncertainty into account in the models, a term (ie, the degree of similarity associated with the normal distribution) was added to the cost function. However, as discussed previously, this term may interfere with the assignment of great importance values to variables by making all *μ* values small. To alleviate this issue, the weight of the term may be lowered, so that the term is less reflected during model training [9,29,33].

However, hyperparameter tuning is not conducted based on a theoretical basis but rather on a heuristic basis. In other words, there is no standard of how many attention layers should be specified and how much the weight should be adjusted for better results. If the goal of building models aims to maximize accuracy, various hyperparameter settings can be tested in the direction of increasing model accuracy. However, there is no clear criterion to maximize the performance of interpretability. In other words, although various hyperparameter settings are tested, finding the best-optimized hyperparameter setting based on the statistical point of view is challenging. Therefore, the variable importance should be understood in a limited way only within the framework of this experiment.

#### Potential Limitations of Interpretability

There was no significant association between the variable importance obtained from the attention mechanism and the effect size obtained from conventional statistics. One of the most probable reasons for this result is that the assumption of the association among input variables is different between conventional methods and deep-learning algorithms. Specifically, conventional statistical methodologies such as linear regression analysis and ANOVA basically estimate effect sizes based on the assumption of independence between input variables [62,63]. Thus, if a particular input variable has nothing to do with an outcome variable, the variable has little effect on the outcome. In contrast, neural network–based algorithms, including deep learning, infer outcome variables by taking into account the dependencies between the input variables [41,42]. Therefore, a variable that is not directly related to an outcome variable but is associated with others that are related to the outcome variable may have a somewhat greater effect on the outcome variable. Owing to these differences, attention results must not be considered to have similar meanings and tendency to the variable effect size from conventional methodologies.

Recent new technologies such as sensors (ie, wearable devices or facilities in operating rooms) have produced new types of data. Since the associations between variables have not yet been fully explored, relying solely on attention mechanisms may lead to a false judgment that variables that have minimal association with the outcome variable are important. Hence, it is advisable to consider the results of attention and conventional statistics together.

Furthermore, in situations where there is a spurious correlation, neither method provides good explanatory power. Spurious correlations can only be eliminated through data preprocessing based on domain knowledge. Hence, care must be taken when implementing both attention models and conventional statistical methods in environments with manifold variables that cannot be preprocessed (ie, included or excluded) using definite knowledge. In particular, finding new features using attention mechanisms may not be adequate in environments where the data are susceptible to spurious correlations owing to a large number of variables but few observations such as in the field of genetic engineering [64,65]. In this case, it may be appropriate to employ results of attention mechanisms for reaffirming existing findings in previous research or supporting informed knowledge.

### Future Direction for Medical Informatics

The results of this study provide several points of guidance for future research in the medical field. First, more empirical evidence should be secured based on various structures in terms of the degree of freedom. It may be desirable to test what attention results are produced when different values of degree of freedom are employed. Particularly, given that the medical field has various data types such as images, natural languages, and numerical values, attention results should be assessed according to the degree of freedom with consideration of the data characteristics [66-69].

Second, attention models with more sophisticated UA should be tested. In this study, the model outcome variable was assumed to depend on the distribution of the attention layer; that is, *P*(*diabetes*|*z*). However, current state-of-the-art Bayesian estimation assumes that the model outcomes depend on all network weights and data; that is, *P*(*outcome*|*z, weight, data*) [9,29]. Thus, it is necessary to evaluate how the variable importance is formed when more up-to-date estimation methods are applied.

Third, more research that strictly evaluates variable importance based on attention mechanisms over diverse disease domains is needed. As found in this study, attention has its limitations in terms of generalizability to conventional statistics and control of spurious correlations. However, since this case study was conducted with a single cohort of Korean patients with diabetes, more empirical evidence from various cohorts or diseases should be tested to confirm that attention mechanisms may not provide any significant meaning. Importantly, for elaborate empirical research, a greater in-depth understanding of the association between covariates and health outcomes is needed. Hence, more domain experts on a specific disease along with data scientists should be actively involved in these studies.

Fourth, methods for controlling the distribution of variable importance should be studied. As revealed in this analysis, the variable importance can be distributed over many variables or concentrated on a few variables depending on the model structure (Figure 7). When examining the overall relationships between covariates and health outcomes such as a comprehensive review of national health status [45-49], it may be desirable to detect many potentially important variables. By contrast, when the relationship between a small number of key variables and outcome is important, such as in the generation of targeted therapy [70,71], the importance should be focused on a few variables. However, to the best of our knowledge, most existing attention studies have not considered the control of the variable importance distribution [8,10-12,24,25,32,33,66,68]. Therefore, more studies on this subject are needed.

### Limitations

There are several limitations to be aware of when assessing the academic value of this study. First, well-behaved data with excellent predictive performance owing to the data characteristics were employed for the analysis. For this reason, the overall AUC performance (see ROC test in Figure 5) might have been good for all approaches (ie, the degree of freedom and UA). When the attention mechanisms are applied to ill-behaved data without manipulation, such as the intentional use of a variable HE_glu as an input variable, the model accuracy may be reduced. If the accuracy of the model is moderate and domain expertise exists for the disease, it is still advisable to attempt a variable importance interpretation. However, if the model accuracy becomes too poor, it may not be worthwhile to interpret the variable importance. Furthermore, categorical variables of both nominal and ordinal types were integerized, and missing values were encoded as the extreme value in this study. Although this operationalization can be efficient in deep-learning algorithms that can learn nonlinear relationships, it is not a robust approach. Thus, it is necessary to identify problems with the approach and to discuss how to deal with them when ill-behaved data with robust operationalization are employed. Furthermore, since data from a single cohort were used, the results of this study, which point out the limitations of the interpretable power of attention mechanisms, should not be generalized. Rather, it should be recognized that accuracy performance and interpretable power may vary depending on the modeling approaches and data.

Second, this study does not guarantee that state-of-the-art estimation methods for UA were applied. Specifically, the models’ outcomes do not depend on network weights. In addition, research on estimation methodologies in deep learning is in progress, and therefore new methodologies are still being developed. Accordingly, the value of this study lies in the framework proposals that suggest the research direction of attention modeling rather than in the details of attention estimation methods.

Third, the design of weights of each local attention layer is not as sophisticated as the design of local attention layers (Figure 4). Specifically, uncertainty considerations are not assumed in the weights layer. Moreover, this layer does not have to be dependent on the local attention layer. In other words, the weights layer may be designed as an independent layer that does not come from the local attention layer. We plan to perform various investigations in this area.

### Conclusions

Attention mechanisms have the potential to make a significant contribution to the medical field, where explanatory power is important, by overcoming the limitations of the noninterpretability of deep-learning algorithms. However, potential problems that may arise when attention mechanisms are applied in practice have not been well studied. Thus, we hope that this study will serve as a cornerstone to raise potential issues, and that many similar studies will be conducted in the future. The cohesive awareness of potential problems arising from attention mechanisms in the field of application will provide theoretical researchers with new goals for problem-solving.

## Appendix

Multimedia Appendix 1 Notations, global attention inference procedure, Herfindahl index values, variable label descriptions, and reparameterization trick concept description.

Multimedia Appendix 2 Codes and algorithms for inferring outcomes.

## Abbreviations

ANOVA: analysis of variance
AUC: area under the curve
LIME: Local Interpretable Model-agnostic Explanations
ROC: receiver operating characteristic
SHAP: Shapley Additive Explanations
UA: uncertainty awareness
