# Supplementary material for: Limitations of Deep Learning Attention Mechanisms in Clinical Research: Empirical Case Study Based on the Korean Diabetic Disease Setting
Source: J Med Internet Res. 2020 Dec 16;22(12):e18418. doi: 10.2196/18418 (PMC7773508; doi:10.2196/18418)
Supplement: Multimedia Appendix 1 [file jmir_v22i12e18418_app1.pdf]

Notation A1. Softmax function

$$\alpha(\gamma)_i = \frac{exp^{\gamma_i}}{\sum_{j=1}^{n} exp^{\gamma_j}}$$

where i and j are node indices, and γ indicates a node value

Notation A2. Cost function of uncertainty awareness models

$$\text{Cost} = -\mathrm{E}_{z\sim q(z|x)}[\log p(y|z)] + D_{KL}(q(z|x)||p(z))$$
$$\mathrm{E}_{z\sim q(z|x)}[\log p(y|z)] = \sum[y\log\hat{y} + (1-y)\log(1-\hat{y})]$$
$$D_{KL}(q(z|x)||p(z)) = \frac{1}{2}\sum[\sigma^2 + \mu^2 - \log(\sigma^2) - 1]$$

where E and D_{KL} are expectation value and Kullback-Leibler divergence, respectively. Kullback-Leibler divergence is a measure of how different one probability distribution is from another [1]. When two distributions are identical, the value of the Kullback-Leibler divergence is 0. The value of Kullback-Leibler divergence increases as two distributions differ more.

Figure A1. Global attention inference procedure

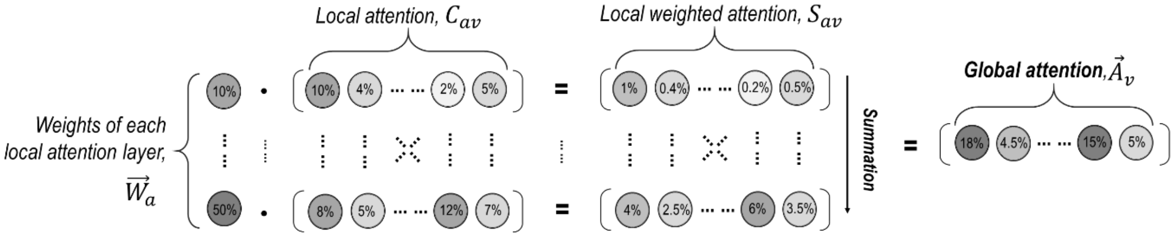

$$\vec{W}_a = \begin{bmatrix} W_1 \\ \vdots \\ W_a \end{bmatrix} \quad , \quad C_{av} = \begin{bmatrix} C_{11} & \cdots & C_{1v} \\ \cdots & \vdots & \cdots \\ C_{a1} & \cdots & C_{av} \end{bmatrix} \quad , \quad S_{av} = \begin{bmatrix} W_1 \cdot C_{11} & \cdots & W_1 \cdot C_{1v} \\ \cdots & \vdots & \cdots \\ W_a \cdot C_{a1} & \cdots & W_a \cdot C_{av} \end{bmatrix} \quad , \quad \vec{A}_v = \left[\sum_{i=1}^{a} S_{a1} \quad \cdots \quad \sum_{i=1}^{a} S_{av} \right]$$

Top: graphical notation; Bottom: mathematical notation

Table A1. Herfindahl index values of fold sets for each model

Reverse rank of fold sets by values	Single	Multi	Single_UA	Multi_UA
10	0.1577	0.0254	0.0097	0.0093
9	0.1914	0.0300	0.0097	0.0104
8	0.1927	0.0323	0.0098	0.0111
7	0.1982	0.0348	0.0098	0.0111
6	0.2345	0.0435	0.0099	0.0111
5	0.2352	0.0465	0.0099	0.0112
4	0.2785	0.0473	0.0099	0.0113
3	0.2816	0.0527	0.0099	0.0115
2	0.2941	0.0806	0.0100	0.0116
1	0.2989	0.0849	0.0100	0.0116

Table A2. Labels of variables selected by importance or effect sizes

**Variable**	**Label (unit)**
age	Age
allownc	Basic Livelihood Security Recipient
BD1	Ever drinked alcohol
BH9_11	Influenza Vaccination
DI5_dg	Diagnosed with myocardial infarction
HE_alt	Alanine aminotransferase (IU/L)
HE_ast	Aspartate aminotransferase test (IU/L)
HE_glu	Fasting blood sugar(mg/dL)
HE_HB	Hemoglobin (g/dL)
HE_HbA1c	Hemoglobin A1C (%)
HE_HBsAg	Hepatitis B surface antigen test
HE_sbp	Systolic blood pressure (mmHg)
HE_THfh1	Diagnosed with thyroid disease (father)
HE_THfh2	Diagnosed with thyroid disease (mother)
HE_THfh3	Diagnosed with thyroid disease (siblings)
HE_Uglu	Urine glucose
HE_Upro	Urine protein
house	Householder
npins	Private health insurance
pa_walk	Walk for 30 minutes at least 5 times a week
sex	Gender
sm_presnt	Current Smoker
wt_itvex	Interview weight value (single year)
wt_pool_1	Interview weight value (combined years)

Concept A1. Reparameterization trick

When training neural network models with probability dependencies on two layers, values of the preceding layer must be sampled from a certain distribution while learning the parameters [2–5]. However, when the normal random sampling technique is applied, the weighted networks are disconnected from each other, so training cannot proceed [2–5]. To solve this issue, a “reparameterization trick” can be used to maintain network continuity while sampling values from the Gaussian distribution. The method generates a latent value z by adding and multiplying the noise sampled from the zero-mean Gaussian to μ and σ^2 respectively [2–5]. That is, $z=\mu(x)+ \sigma^2(x)\times\epsilon$, $\epsilon \sim N(0,1)$

References

1. Kullback S, Leibler RA. On Information and Sufficiency. Ann Math Stat 1951 Mar;22(1):79–86. [doi: 10.1214/aoms/1177729694]

2. Heo J, Lee HB, Kim S, Lee J, Kim KJ, Yang E, Hwang SJ. Uncertainty-aware attention for reliable interpretation and prediction. 2018. p. 909–918.

3. Ehsan Abbasnejad M, Dick A, van den Hengel A. Infinite variational autoencoder for semi-supervised learning. 2017. p. 5888–5897.

4. Kingma DP, Welling M. Auto-encoding variational bayes. ArXiv Prepr ArXiv13126114 2013;

5. Kingma DP, Salimans T, Welling M. Variational dropout and the local reparameterization trick. 2015. p. 2575–2583.
