# Supplementary material for: Limitations of Deep Learning Attention Mechanisms in Clinical Research: Empirical Case Study Based on the Korean Diabetic Disease Setting
Source: J Med Internet Res. 2020 Dec 16;22(12):e18418. doi: 10.2196/18418 (PMC7773508; doi:10.2196/18418)
Supplement: Multimedia Appendix 2 [file jmir_v22i12e18418_app2.pdf]

Code A1. Keras codes for basic attention

```
InputLayer = Input(shape=(NDimenIn,))
AttentionLayer = Dense(NDimenIn, activation='softmax')( InputLayer)
ContextLayer = dot([AttentionLayer, InputLayer],axes=[1,1])
Outcome = Lambda(lambda x:keras.backend.sum(x, axis=-1, keepdims=True))(ContextLayer)
Outcome = Activation('sigmoid')( Outcome)

BasicAttention = Model(inputs=InputLayer, outputs= Outcome)
```

NDimenIn is the number of input variables

Code A2. Keras codes for single attention

```
InputVec = Input(shape=(NDimenIn,))
InpNormalize = BatchNormalization()(InputVec)
Dense1 = Dense(NDimenIn, activation='tanh')(InpNormalize)
Dense1Drop = Dropout(0.1)(Dense1,training=True)
Attention = Dense(NDimenIn, activation='softmax')(Dense1Drop)
OutDense = dot([Attention, InputVec],axes=[1,1])
OutDense = BatchNormalization()(OutDense)
OutDense = Activation('sigmoid')(OutDense)
```

NDimenIn is the number of input variables

Code A3. Keras codes for single attention with uncertainty awareness

```
class Reparameterization(Layer):

    def __init__(self):
        super(Reparameterization, self).__init__()

    def call(self, args):
        Mu, logVar = args
        epsilon = K.random_normal(shape=(K.shape(Mu)[0], int(Mu.shape[1])),mean=0., stddev=1)
        return Mu + K.exp(logVar) * epsilon

def TotalLoss (y_true, y_pred):
            return K.mean(losses.binary_crossentropy(y_true, y_pred) + KL_loss)

InputVec = Input(shape=(NDimenIn,))
InpNormalize = BatchNormalization()(InputVec)
Dense1 = Dense(NDimenIn, activation='tanh')(InpNormalize)
Dense1Drop = Dropout(0.1)(Dense1,training=True)
Mu = Dense(NDimenIn)(Dense1Drop)
logVar = Dense(NDimenIn, activation='softplus')(Dense1Drop)
LatentVec = Reparameterization()([Mu, logVar])
Attention = Activation(activation='softmax')(LatentVec)
OutDense = dot([Attention, InputVec],axes=[1,1])
OutDense = BatchNormalization()(OutDense)
OutDense = Activation('sigmoid')(OutDense)

LamRatio = 1
KL_loss = 1 + logVar - K.square(Mu) - K.exp(logVar)
KL_loss = -0.5*K.mean(KL_loss, axis=-1)
KL_loss *= LamRatio
```

NDimenIn is the number of input variables

Code A4. Keras codes for multi-attention

```
InputVec = Input(shape=(NDimenIn,))
InpNormalize = BatchNormalization()(InputVec)
Dense1 = Dense(NDimenIn, activation='tanh')(InpNormalize)
Dense1Drop = Dropout(0.1)(Dense1,training=True)
AttList = []
for Attidx in range(AttLaySize):
    Attention = Dense(NDimenIn, activation='softmax')(Dense1Drop)
    Attention = Reshape((1,NDimenIn))(Attention)
    AttList.append(Attention)
AttentionWeightMerged = concatenate(AttList,axis=1)
ContextLayer = dot([AttentionWeightMerged, Reshape((1,NDimenIn))(InputVec)], axes=[2,2])
FlattDense = Flatten()(ContextLayer)
Dense2 = Dense(AttLaySize, activation='sigmoid')(FlattDense)
Dense2Normal = BatchNormalization()(Dense2)
ModelWAtt = Dense(AttLaySize, activation='softmax')(Dense2Normal)

OutDense = dot([FlattDense, ModelWAtt], axes=[1,1])
OutDense = BatchNormalization()(OutDense)
OutDense = Activation('sigmoid')(OutDense)
```

NDimenIn is the number of input variables; AttLaySize is the number of local attention layers

Code A5. Keras codes for multi-attention with uncertainty awareness

```
class Reparameterization(Layer):

        def __init__(self):
                super(Reparameterization, self).__init__()

        def call(self, args):
                Mu, logVar = args
                epsilon = K.random_normal(shape=(K.shape(Mu)[0], int(Mu.shape[1]),
int(Mu.shape[2])),mean=0., stddev=1)
                return Mu + K.exp(logVar) * epsilon

def TotalLoss (y_true, y_pred):
                return K.mean(losses.binary_crossentropy(y_true, y_pred) + KL_loss)

InputVec = Input(shape=(NDimenIn,))
InpNormalize = BatchNormalization()(InputVec)
Dense1 = Dense(NDimenIn, activation='tanh')(InpNormalize)
Dense1Drop = Dropout(0.1)(Dense1,training=True)

MuList = []
logVarList = []

for Attidx in range(AttLaySize):
   Mu = Dense(NDimenIn)(Dense1Drop)
   Mu = Reshape((1,NDimenIn))(Mu)
   MuList.append(Mu)

   logVar = Dense(NDimenIn, activation='softplus')(Dense1Drop)
   logVar = Reshape((1,NDimenIn))(logVar)
   logVarList.append(logVar)

MuMerged = concatenate(MuList,axis=1)
logVarMerged = concatenate(logVarList,axis=1)
LatentVec = Reparameterization()([MuMerged, logVarMerged])

Attention = Activation(activation='softmax')(LatentVec)
ContextLayer = dot([Attention, Reshape((1,NDimenIn))(InputVec)], axes=[2,2])
FlattDense = Flatten()(ContextLayer)
Dense2 = Dense(AttLaySize, activation='sigmoid')(FlattDense)
Dense2Normal = BatchNormalization()(Dense2)
ModelWAtt = Dense(AttLaySize, activation='softmax')(Dense2Normal)

OutDense = dot([FlattDense, ModelWAtt], axes=[1,1])
OutDense = BatchNormalization()(OutDense)
OutDense = Activation('sigmoid')(OutDense)


LamRatio = 1
KL_loss = 1 + logVarMerged -    K.square(MuMerged) - K.exp(logVarMerged)
KL_loss = Reshape((NDimenIn*AttLaySize,))(KL_loss)
KL_loss = -0.5*K.mean(KL_loss, axis=-1)
KL_loss *= LamRatio
```

NDimenIn is the number of input variables; AttLaySize is the number of local attention layers

Algorithm A1. Pseudocode for inferring outcome

```
Start procedure
Start For loop (size = 100) # Conducting Monte Carlo simulation
        Fit a model to predict ① probabilities (1=diabetes, 0=non-diabetes)
        Store ① in probability list
End For loop
Average probability list along individuals
End procedure
```

Green characters indicate typical programming algorithms; text in brown after # indicates a comment; italic characters indicate variables.

Algorithm A2. Pseudocode for inferring attention values of single attention models

```
Start procedure
Start For loop (size = 100) # Conducting Monte Carlo simulation
        Fit a model to predict ① attentions
        Average ① along individuals → ② attention at iteration levels # shape: (variables)
        Store ② in attention list
End For loop
Average attention list along individuals # mean attention values at model levels
End procedure
```

Green characters indicate typical programming algorithms; text in brown after # indicates a comment; italic characters indicate variables.

Algorithm A3. Pseudocode for inferring attention values of multi-attention models

```
Start procedure
Start For loop (size = 100) # Conducting Monte Carlo simulation
        Fit a model to predict both ① local attentions and ② weights of local attentions
        Multiply ② by the corresponding vector of ① → ③ local weighted attentions
        Sum ③ along variables → ④ global attention # shape: (individual, variables)
        Average ④ along individuals → ⑤ global attention at iteration levels
                                                        # shape: (variables)
        Store ⑤ in global attention list
End For loop
Average global attention list along individuals # mean attention values at model levels
End procedure
```

Green characters indicate typical programming algorithms; text in brown after # indicates a comment; italic characters indicate variables.
